# Longitudinal Relationships between Asthma-Specific Quality of Life and Asthma Control in Children; The Influence of Chronic Rhinitis

**DOI:** 10.3390/jcm9020555

**Published:** 2020-02-18

**Authors:** Dillys van Vliet, Brigitte A. Essers, Bjorn Winkens, Jan W. Heynens, Jean W. Muris, Quirijn Jöbsis, Edward Dompeling

**Affiliations:** 1 Department of Paediatric Pulmonology, School for Public Health and Primary Care (CAPHRI), Maastricht University Medical Centre (MUMC^+^), 6202 AZ Maastricht, The Netherlands; dillysv@hotmail.com (D.v.V.); r.jobsis@mumc.nl (Q.J.); 2 Department of Clinical Epidemiology and Medical Technology Assessment, MUMC^+^, 6229 HX Maastricht, The Netherlands; brigitte.essers@mumc.nl; 3 Department of Methodology and Statistics, CAPHRI, MUMC^+^, 6229 HA Maastricht, The Netherlands; bjorn.winkens@maastrichtuniversity.nl; 4 Department of Paediatrics, Zuyderland Medical Centre, 6162 BG Sittard-Geleen, The Netherlands; jan.heijnens@mumc.nl; 5 Department of Primary Care Medicine, CAPHRI, MUMC^+^, 6229 HA Maastricht, The Netherlands; jean.muris@maastrichtuniversity.nl

**Keywords:** asthma, asthma-specific quality of life, chronic rhinitis, disease-specific quality of life, health-related quality of Life (HRQLQ), children, longitudinal study

## Abstract

Managing pediatric asthma includes optimizing both asthma control and asthma-specific quality of life (QoL). However, it is unclear to what extent asthma-specific QoL is related to asthma control or other clinical characteristics over time. The aims of this study were to assess in children longitudinally: (1) the association between asthma control and asthma-specific QoL and (2) the relationship between clinical characteristics and asthma-specific QoL. In a 12-month prospective study, asthma-specific QoL, asthma control, dynamic lung function indices, fractional exhaled nitric oxide, the occurrence of exacerbations, and the use of rescue medication were assessed every 2 months. Associations between the clinical characteristics and asthma-specific QoL were analyzed using linear mixed models. At baseline, the QoL symptom score was worse in children with asthma and concomitant chronic rhinitis compared to asthmatic children without chronic rhinitis. An improvement of asthma control was longitudinally associated with an increase in asthma-specific QoL (*p*-value < 0.01). An increased use of β_2_-agonists, the occurrence of wheezing episodes in the year before the study, the occurrence of an asthma exacerbation in the 2 months prior to a clinical visit, and a deterioration of lung function correlated significantly with a decrease in the Pediatric Asthma Quality of Life Questionnaire (PAQLQ) total score (*p*-values ≤ 0.01). Chronic rhinitis did not correlate with changes in the PAQLQ score over 1 year. The conclusion was that asthma control and asthma-specific QoL were longitudinally associated, but were not mutually interchangeable. The presence of chronic rhinitis at baseline did influence QoL symptom scores. β_2_-agonist use and exacerbations before and during the study were inversely related to the asthma-specific QoL over time.

## 1. Introduction

Respiratory symptoms have a significant influence on the daily life of children with asthma [1,2]. In the management of asthma, monitoring of both the asthma control and the quality of life (QoL) is important. International guidelines mention optimal QoL as an important objective in asthma management, but offer no guidance on how or when to base clinical decisions on asthma-specific QoL [3,4,5].

As guidelines recommend medication titration predominantly based on asthma control, additional knowledge about the longitudinal relationship between pediatric asthma control and asthma-specific QoL is important [3,4,5]. Studies focusing on asthma-specific QoL found a fair to good cross-sectional association between the QoL and the asthma level of disease control in children [1,6,7,8,9,10,11,12]. However, several important questions remain. First, as asthma is a chronic disease, it is relevant to know whether changes in the quality of life over the course of time are correlated with changes in the asthma control. To date, longitudinal data on this topic are lacking [1,6,7,8,9,10,11,12,13]. 

Second, there is no information available about the course and variation of asthma-specific QoL in children. This information may give insight into the frequency with which asthma-specific QoL should be monitored in clinical care. Third, as similar questions were used to assess the asthma control and the QoL in some studies, it is unclear to what extent this may have over-exaggerated the correlation between the asthma control and the QoL [9,12]. Fourth, very little is known about the longitudinal relationship between asthma-specific QoL and clinical characteristics, such as the presence of chronic rhinitis, the use of rescue medication, the daily dosage of inhaled corticosteroids, the exacerbation rate, lung function, atopy, airway inflammation (e.g., fractional exhaled nitric oxide (FeNO) levels), and bronchial hyperresponsiveness. 

Although it is generally assumed that these clinical characteristics affect the QoL, there are little longitudinal data in children to confirm this. 

Therefore, the objectives of this study are to determine the association over time:

1) between asthma control and asthma-specific QoL;
2) between clinical characteristics (e.g., the presence of chronic rhinitis, daily dosage of inhaled corticosteroids, lung function impairment, use of rescue medication, FeNO, and the occurrence of asthma exacerbations) and asthma-specific QoL.

## 2. Methods

### 2.1. Study Design and Patients

Children with persistent asthma aged 6–17 years were included in this one-year longitudinal cohort study (clinicaltrial.gov NCT 01239238) as described previously [14]. Patients were recruited at the outpatient clinics of 2 clinical centers (Sittard and Maastricht) in the Netherlands. All asthmatic children were treated at the outpatient clinic of these 2 specialized pediatric pulmonology centers for at least 6 months and had received inhaled corticosteroids (ICS) in the year preceding the study. All children met the Global Initiative for Asthma (GINA) criteria and the following criteria of the Dutch Society of Pediatrics for an asthma diagnosis: (1) recurrent episodes of wheezing, coughing, breathlessness, or chest tightness [3,5]; (2) reversibility to a bronchodilator defined as an increase in the forced expiratory volume in 1 s (FEV_1_) of ≥ 9% of the predicted value [5,15]; and/or (3) bronchial hyperresponsiveness to histamine defined as a 20% drop in the FEV_1_ after inhalation of histamine ≤ 8 mg/mL [5]. Patients were excluded in the case of cardiac abnormalities, mental retardation, congenital abnormalities or existence of a syndrome, active smoking, immunotherapy, or no technical satisfactory performance of lung function measurements. 

For this study, ethical approval was obtained by the Medical Ethical Committee of the Maastricht University Medical Centre (NL33101.068.10/METC 10-2-064). All parents and children aged twelve years and older signed an informed consent form before the start of the study. All methods and measurement were performed in accordance with the relevant guidelines and regulations.

### 2.2. Study Parameters

Every 2 months, regular outpatient visits at the hospital took place. During these clinical visits, measurements of asthma control, lung function, FeNO and asthma-specific QoL were taken by a trained research nurse or a medical doctor.

### 2.3. Questionnaires on Asthma Control, Asthma-Specific QoL, and the International Study of Asthma and Allergies in Childhood (ISAAC)

Asthma control was assessed using 2 methods: (1) an Asthma Control Questionnaire (ACQ) and (2) the GINA criteria [16,17]. The following cut-off points for the level of control were used: ACQ≤0.75 controlled asthma, 0.75< ACQ ≤1.5 partly controlled, and ACQ >1.5 uncontrolled asthma. In addition, the asthma control level during the previous 2 weeks was assessed by scoring symptoms in combination with a FEV_1_ assessment, as recommended in the GINA—Asthma Management and Prevention-guidelines [3]. 

To assess the asthma-specific QoL, children completed the Pediatric Asthma Quality of Life Questionnaire (PAQLQ) [8,11,18]. The standardized version of the PAQLQ contains 23 questions in 3 domains, i.e., activity limitations, symptoms, and emotional function. The range in scores is from 1–7, which represents poor to good asthma-specific QoL [8]. All children completed the ACQ and PAQLQ by themselves, so interference of parents was avoided as much as possible. 

### 2.4. FeNO

Children performed a FeNO online measurement using a NIOX analyzer (NIOX MINO®, Aerocrine, Solna, Sweden) according to American Thoracic Society (ATS) and European Respiratory Society (ERS) standards [19]. 

### 2.5. Dynamic Spirometry and Reversibility

Patients were instructed to stop short-acting bronchodilators at least 8 h, and long-acting bronchodilators at least 48 h, before measurement. First, dynamic spirometry was performed by means of the ZAN 100^®^ spirometer, according to ATS/ERS standards (nSpire Health GmbH, Oberthulba, Germany) [20]. The highest value of 3 correctly performed maximal expiratory flow-volume (MEFV) curves was used for analysis. The recorded parameters included the FEV_1_, forced vital capacity (FVC), and maximum expiratory flow at 50% of FVC (MEF_50_), all expressed as a percentage of the predicted value. Second, the patient inhaled 400 µg of salbutamol, and after 15 m lung function measurements were repeated in order to test for reversibility to a bronchodilator. 

### 2.6. Bronchial Hyperresponsiveness

Bronchial hyperresponsiveness at baseline was evaluated by the histamine challenge test [21]. At first, an aerosol of buffered saline was inhaled, followed by aerosols of histamine solutions with a doubling of the concentration from 0.03 to 16 mg/mL at intervals of 5 m. The FEV_1_ was measured at 30, 90, and 120 s after completed inhalation. The percentage decline in the FEV_1_ was calculated and the test was stopped when a drop of 20% in the FEV_1_ occurred (PC_20_), or the highest concentration of 16 mg/mL was administered. The PC_20_ threshold was calculated from a log concentration versus dose response curve. After reaching the threshold, children inhaled 800 µg of salbutamol, and then 3 MEFV curves were performed. 

### 2.7. Atopy

Sensitization to allergens was objectified by the Phadiatop^®^ (Phadia, Uppsala, Sweden), RAST^®^ (Pharmacia, Uppsala, Sweden) or allergen skin test. These tests were performed preceding the study or at baseline.

### 2.8. Medication Titration

Patients were treated according to the GINA guidelines [3]. Medication titration was performed on the basis of asthma control levels based on the GINA criteria (Table 1).

### 2.9. Definition of Exacerbation

The definition of an asthma exacerbation was based on the latest ATS/ERS guidelines [22]. 

### 2.10. ISAAC Questionnaire and Chronic Rhinitis

At baseline, parents completed electronically the ISAAC (International Study of Asthma and Allergies in Childhood) questionnaire [23]. The items focusing on asthma symptoms were used for statistical analysis. The ISAAC questionnaire was completed by 94% of all parents. A child was considered to have chronic rhinitis in the case of a positive response to 2 ISAAC questions (‘chronic rhinitis without a cold’ and ‘chronic rhinitis in the past 12 months’). 

### 2.11. Exposure to Second-Hand Smoke 

Parental smoking was assessed using an electronic questionnaire, which was completed by 94% of all parents. Passive smoking or exposure to second-hand smoke was defined as smoking of one or both parents in the presence of the child. 

### 2.12. Data Collection

The collected data were checked and cleaned by an independent monitoring board (Clinical Trial Centre Maastricht). All recorded data during visits were stored in a secured database. In addition, all electronic questionnaires were completed at home. 

### 2.13. Data Analysis

The description of baseline characteristics occurred as follows: numerical variables were expressed as the mean and standard deviation (SD) or as the median and interquartile ranges (IQR, i.e., 25th–75th percentile) and categorical variables were expressed as numbers and percentages. The variation of baseline PAQLQ scores for different asthma control levels was described as the mean and standard deviation. In addition, at baseline the coefficient of variation was calculated for each asthma control level (CV = mean/standard deviation). 

To control for interdependency between repeated measurements in the same participant, linear mixed models were used [24]. All participants were included in the analysis, including children who dropped out during the study. In the mixed models, the PAQLQ total scores and, thereafter, the domains were included as the dependent variable. First, the course of the PAQLQ scores during the study was tested for significance in a simple model without the correction of any clinical variable. Second, in order to analyze the association between asthma control and the PAQLQ scores, the ACQ and GINA asthma control levels were included as an independent variable separately. Third, the clinical characteristics, FEV_1_ percentage of the predicted value, and β_2_-agonist use were included in a new model, while asthma control was excluded. Asthma symptoms represent 1 domain of the PAQLQ; therefore, symptoms were not included as a separate independent variable in any model. Besides, in all models the age, sex, trial site, season, bronchial hyperresponsiveness, atopy, exposure to second-hand smoke, FeNO, and the inhaled daily dose of corticosteroids were included as independent variables. The *p*-values < 0.05 were considered significant. Data were analyzed using SPSS 20 (SPSS Inc., Chicago, IL, USA). 

## 3. Results

### 3.1. Patient Characteristics

A total number of 331 children with doctor-diagnosed asthma were asked to participate in the study, of which 96 children actually participated [14]. The majority of subjects were atopic and had severe bronchial hyperresponsiveness despite the use of a moderate daily dose of inhaled corticosteroids. Active parental smoking was reported in the cases of 26% of the children, whereas exposure to second-hand smoke was reported in the cases of 8% of the children (Table 2). There were no clinical important differences in the baseline characteristics between the two centers.

### 3.2. Influence of Chronic Rhinitis on QoL at Baseline

In comparison with children without chronic rhinitis (*n* = 27), children with rhinitis (*n* = 62) had a lower PAQLQ symptoms domain score (mean score (SD) of 5.8 (1.1) versus a score of 6.3 (0.6), *p* = 0.016), a comparable PAQLQ activity limitation score (mean (SD) of 5.9 (1.0) versus 6.1 (0.7), *p* = 0.352) or a PAQLQ emotional domain (mean (SD) of 6.5 (1.0) versus 6.6 (0.6), *p* = 0.516), and a tendency towards a lower PAQLQ total score (mean (SD) of 6.1 (1.0) versus 6.4 (0.5), *p* = 0.076). A lower PAQLQ score indicates a worse quality of life. 

### 3.3. Course and Variability of Asthma-Specific QoL during the Study

**Figure 1:**
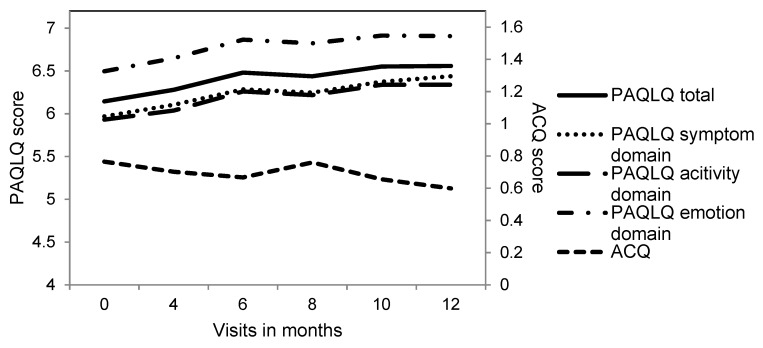
Overview of the course of the PAQLQ and ACQ scores during 12 months. On the right-hand side, the scale and numbers of the ACQ score are given, on the left-hand side, the scale and numbers of the PAQLQ score.

**Figure 2:**
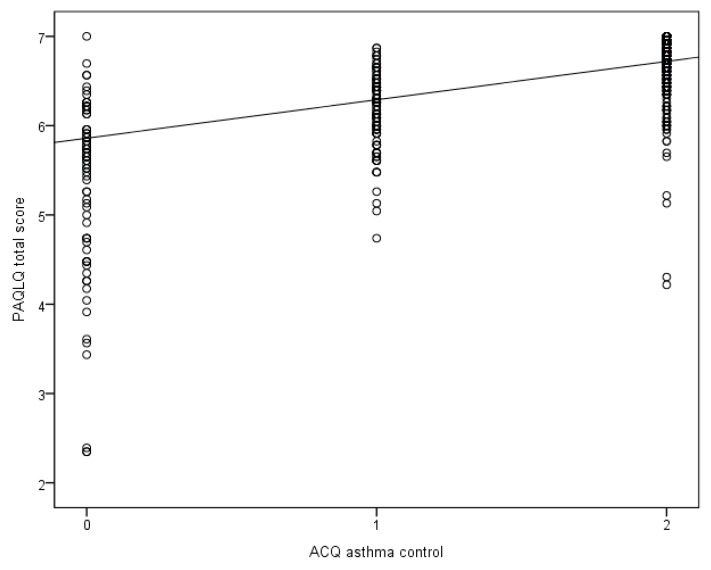
Associations between ACQ asthma control and PAQLQ total scores in a linear mixed model―all measurements taken during one year were included. The regression line is based on a model in which the ACQ level of asthma control is included; the estimates of this model are given in Table 3. For factors in the equation other than ACQ asthma control, mean values were used. ACQ asthma control: 0 = uncontrolled, 1 = partly controlled, and 2 = controlled.

Overall asthma-specific QoL and asthma control improved during the year (*p*-value < 0.01) (Figure 1). The mean level of the symptoms and activity limitations domain was lower than the level of the emotional functioning domain (Figure 1). In 46% of the children, this emotional functioning domain at baseline showed a maximum score of seven. The variability of the PAQLQ total scores at baseline was larger in the partly or uncontrolled children (coefficient of variation, CV = 19%), than in the controlled children (CV = 6%). This variability is shown in Figure 2, where all the measurements of all the children are included. 

### 3.4. Association between Asthma Control and PAQLQ Scores during a One Year Follow-up

Asthma control based on the ACQ had the strongest longitudinal association with the PAQLQ total scores compared to the occurrence of wheezing episodes in the preceding year and the occurrence of an exacerbation in the previous 2 months (*p*-value (estimate); <0.01 (0.43)) (Table 3). The estimate of 0.43 means that an improvement of asthma control from uncontrolled to partly controlled, or from partly controlled to controlled disease, resulted in an increase of the PAQLQ total score of 0.43. Therefore, deterioration of asthma control from controlled to uncontrolled was associated with a clinically relevant decrease in the PAQLQ total score of 0.86. Additionally, with the PAQLQ subdomains, asthma control had the strongest association of all the clinical characteristics. The estimates for the subdomains were highest for the symptoms and activity limitation and small but significant for the emotional functioning domain (*p*-value (estimate) (95% CI); <0.01 (0.05) (0.03, 0.08)).

We also performed an analysis with asthma control according to the GINA criteria. Similarly to asthma control based on the ACQ, a strong longitudinal association with the PAQLQ total score and a comparable pattern for the PAQLQ domains was found (Table 4). Additionally, the occurrence of wheezing episodes in the preceding year was associated with the PAQLQ total score. Of all the PAQLQ domains, the emotional functioning domain had the smallest association with asthma control based on the GINA criteria (*p*-value (estimate) (95% CI); <0.01 (0.09) (0.05, 0.13). All models were adjusted for clinical characteristics, as described in the method section.

### 3.5. Factors Independently Related to PAQLQ Scores during One Year Follow-up

**Figure 3:**
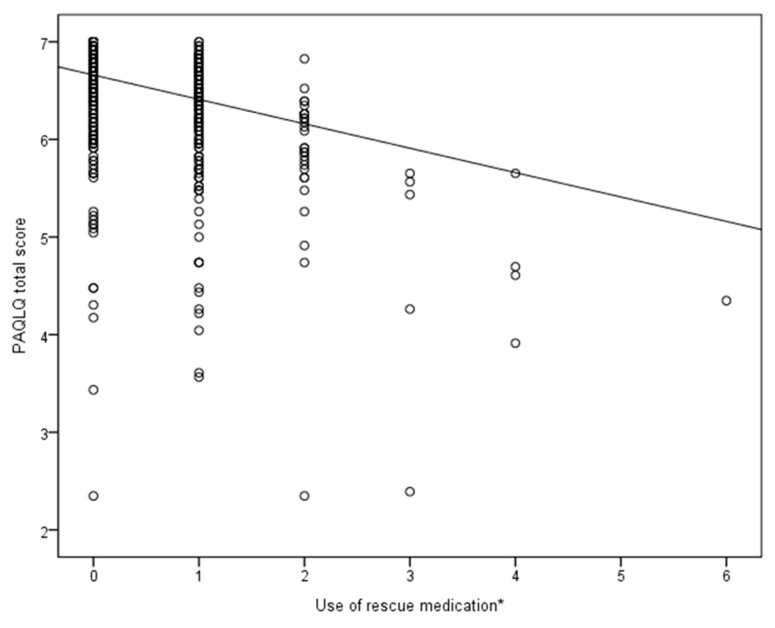
Associations between β_2_-agonist use and the PAQLQ total score in a linear mixed model―all measurements taken during one year are included^†^. * Use of rescue medication during the week preceding the clinical visit: 0 = 0 puffs most days, 1 = 1–2 puffs most days, 2 = 3–4 puffs most days, 3 = 5–8 puffs most days, 4 = 9–12 puffs most days, 5 = 13–16 puffs most days, and 6=more than 16 puffs most days. The regression line is based on the model in which the asthma control levels were excluded, the estimates of this model are given in Table 4. For factors in the equation other than the use of rescue medication, mean values were used.

**Figure 4:**
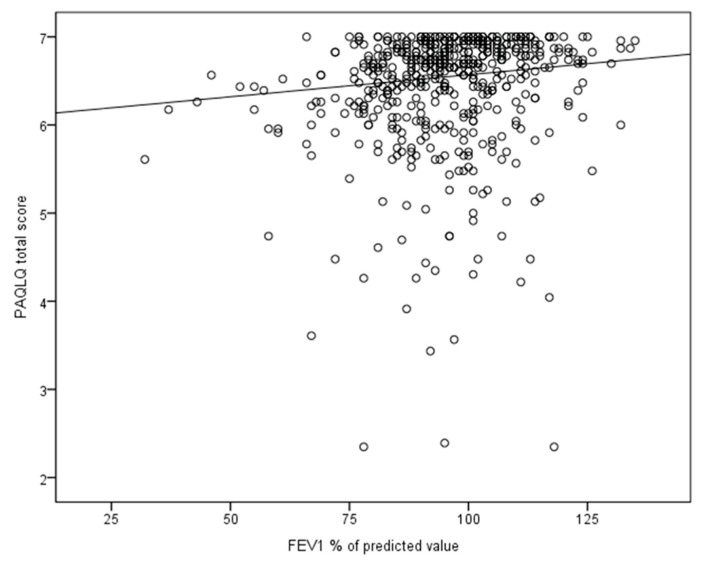
Associations between lung function and the PAQLQ total score in a linear mixed model―all measurements taken during one year were included. The regression line is based on the model in which the asthma control levels were excluded, the estimates of this model are given in Table 5. For factors in the equation other than the FEV_1_ percentage of the predicted value, mean values were used.

Finally, a model consisting of all predefined clinical characteristics (except the ACQ or GINA asthma control) showed that three factors were independently associated with a decrease in the PAQLQ total scores: (1) an increased use of β_2_-agonists (Table 5 and Figure 3), (2) the occurrence of wheezing episodes in the year preceding the study, and (3) the occurrence of an asthma exacerbation in the 2 months preceding the PAQLQ assessments (Table 5). Although the FEV_1_ percentage of the predicted value was also significantly associated with the PAQLQ total score, the estimates were small (*p*-value (estimate); <0.01 (0.05)) (Table 5, Figure 4). The daily dose of inhaled corticosteroids and FeNO levels showed no longitudinal association with the PAQLQ scores (Table 5). In contrast to the model in which the asthma control was included, no factor in this model was associated with the PAQLQ emotional functioning domain. The presence of chronic rhinitis was not associated with changes in the PAQLQ total score or subdomain scores. 

## 4. Discussion

This study showed that, at baseline, the presence of chronic rhinitis in the children with asthma was significantly related to a lower QoL symptom score and a tendency towards a lower QoL total score. Deterioration of asthma control was associated with a clinically relevant decrease in the pediatric asthma-specific QoL (PAQLQ) score during 12 months. In addition, the level of asthma control, as assessed by the ACQ and according to the GINA criteria, were to the same extent independently related to asthma-specific QoL. Moreover, we found that an increase in β_2_-agonist use, the occurrence of an exacerbation in the previous 2 months, and the occurrence of wheezing episodes in the year preceding the study were associated with a decrease in pediatric asthma-specific QoL. Of all domains, asthma control was most strongly associated with the symptoms and activity limitations domain and weakly with the emotional functioning domain of the PAQLQ. The presence of chronic rhinitis was not related to changes in PAQLQ scores during one year. 

This prospective study showed that β_2_-agonist use, the occurrence of an exacerbation in the previous 2 months and the occurrence of wheezing episodes in the preceding year were related to pediatric asthma-specific QoL. Lung function impairment as assessed by the FEV_1_ percentage of the predicted value had a weak association with asthma-specific QoL. This finding is in line with a meta-analysis which determined the degree of association between the lung function and subjective measures in 8994 children and adults from 27 randomized controlled trials [25]. In this meta-analysis, a moderate correlation between the lung function and asthma-specific QoL at time points 0 and 12 weeks was found [25]. Moreover, we found no association between a measure of airway inflammation (FeNO) and asthma-specific QoL. This finding is consistent with the data of De Jongste et al. in a study of 151 children with atopic asthma, in which medication was titrated based on FeNO plus symptom telemonitoring or symptom monitoring only [26]. Daily FeNO monitoring did not improve asthma control or asthma-specific QoL completed by caregivers [26]. 

In our cohort, asthma exacerbations were associated with a decrease in asthma-specific QoL. This is supported by Luskin et al., who showed that a greater severity and number of exacerbations were associated with a decrease in asthma-specific QoL [27]. Furthermore, the association between the asthma level of control and asthma-specific QoL we found is consistent with other studies focusing on asthma-specific QoL in children [1,6,7,8,9,10,11,12,27,28,29,30,31,32]. It is known that chronic rhinitis in children with asthma is not only frequently occurring but also an underestimated problem by both patients, parents, and doctors [3]. Indeed, at baseline, we found a negative influence of chronic rhinitis on the QoL symptom scores although no clear effect of chronic rhinitis in the longitudinal analysis was found. This stresses the importance of the proper treatment of chronic rhinitis as this probably will affect asthma control and QoL in a positive way [3]. 

A strength of our prospective one-year study is that it provides insight into the longitudinal associations between clinical characteristics and asthma-specific QoL. This is valuable information because asthma is a chronic disease [28]. Furthermore, the PAQLQ questionnaire in this study was completed by children and not by their parents. This is preferable because previous studies have shown that parents are not able to indicate the quality of life of their child [30,31]. A limitation of our study may have been the absence of additional demographic parameters. However, we predefined a factor with a negative influence on asthma that is associated with social economic status, namely exposure to second-hand smoke. Another limitation may have been the relatively high emotional functioning domain scores at baseline. This may limit the possibility of finding associations with other variables over time. However, in this study, a significant increase in the PAQLQ total scores was demonstrated and significant associations with asthma control were found for this total score and the other domains. Moreover, although there was no power or sample size calculation performed for this explorative study, there was a significant result for the primary research question (a significant association between ACQ and PAQLQ over time) This indicates that the study was not underpowered for detecting a significant effect for the primary endpoints. 

How can the findings of our study be explained? First, there was a close association between asthma control and asthma-specific QoL, suggesting that the actual level of disease control determined, to a large extent, the perceived disease-specific QoL. However, we also found that asthma control and asthma-specific QoL were not mutually interchangeable. These variables reflect two different aspects of asthma as shown in Figure 2; at a certain level of asthma control, there was still a considerable variation in levels of asthma-specific QoL, even in children with controlled asthma. From our study it was unclear what caused this variation and discrepancy. Other researchers found evidence that emotional/psychological factors influence the asthma-specific QoL [13,26]. In a longitudinal observational study in patients of 13 years and over, the association between asthma triggers and asthma-specific QoL was investigated [27]. Of all triggers, emotional stress was most strongly associated with asthma-specific QoL, which may explain part of the variation in QoL at a certain asthma control level. Second, the increase in the PAQLQ total scores in this study could be a study effect, which may be explained by the strict titration of asthma medication based on the GINA criteria every 2 months. This explanation was supported by our finding that asthma control levels also improved during the 12-month study period and these were independently related to the PAQLQ total scores. Third, we found that wheezing episodes in the year preceding the study, the occurrence of exacerbations in the 2 months prior to the clinical visit, and the use of a β_2_-agonist were related to a lower asthma-specific QoL. These findings confirm that exacerbations have significant impact on the experienced well-being of children with asthma. It is known that exacerbations often require urgent care or even emergency care visits and are a source of anxiety and uncertainty in both children and parents [3]. More use of β_2_-agonists are linked to an increase in asthma symptoms, to limitations in physical activity, and possibly to extra clinical visits [3]. This appeared to have a negative influence on asthma-specific QoL in this longitudinal study. Fourth, in contrast to the use of β_2_-agonists, we found no substantial influence of the daily dose of inhaled corticosteroids on asthma-specific QoL. This is probably because the ICS dose was more closely correlated to the chronic course of the disease and has no direct symptom-relieving effect, whereas β_2_-agonists are more closely related to acute increases in disease activity and have a direct effect on symptoms. Moreover, the side effects of ICS are often mild. 

The findings of our study imply that, in pediatric asthma, the level of asthma control or the presence of other clinical characteristics cannot be used as a substitute for asthma-specific QoL. We found an association between asthma control based on the ACQ and all PAQLQ domains. However, some children with controlled asthma had a relatively low asthma-specific QoL (Figure 2). This suggests that the measurement of asthma-specific QoL in children during clinical contact in addition to asthma control may be valuable, because it may reveal, in some cases, a relatively low QoL while a child has controlled asthma. 

In conclusion, a strong longitudinal relationship between pediatric asthma-specific QoL and asthma control during a 12 month follow-up study was found. The presence of chronic rhinitis at baseline was associated with a worse QoL. Moreover, an increase of β_2_-agonist use, the occurrence of wheezing episodes in the year preceding the study, and the occurrence of an asthma exacerbation during the study were negatively related to asthma-specific QoL in children. 

## Figures and Tables

**Table 1:** Criteria for medication titration (step up, no change, or step down).

**Step up**
Uncontrolled asthma in 1 visit, or
partly controlled asthma during 2 consecutive visits.
**No change in treatment**
One visit with controlled asthma, or
one visit with partly controlled asthma.
**Step down**
Two consecutive visits with controlled asthma.

**Table 2:** Characteristics of the total population at baseline (*n* = 96).

Parameter	Value
Mean age [range], in years	10 [6–17]
Sex male/female, *n*	50/46
ACQ score, median [IQR]	0.6 [0.3–1]
PAQLQ total score, median [IQR]	6.4 [5.9–6.7]
Symptoms domain, median [IQR]	6.4 [5.5–6.7]
Activity limitations domain, median [IQR]	6.2 [5.3–6.6]
Emotional functioning domain, median [IQR]	6.9 [6.5–7.0]
FeNO, ppb: median [IQR]	12.5 [8.0–31.0]
FEV_1_ % predicted, mean ± SD	96.8 ± 14.2
Reversibility, increase in FEV_1_ % predicted: mean ± SD	6.6 ± 8.5
ICS dose of inhaled fluticasone or equivalent, mean ± SD *	269 ± 175
PC_20_, mg/mL: median [IQR] ^†^	1.2 [0.3–2.9]
Atopic, n % ^‡^	76
Chronic rhinitis, %	70
Wheezing episodes past year, %	58
Parental smoking, %	26
Exposure to second-hand smoke, %	8

ACQ = Asthma Control Questionnaire, IQR = interquartile range, PAQLQ = Pediatric Asthma Control Quality of Life Questionnaire, FeNO = fractional exhaled nitric oxide, FEV_1_: forced expiratory volume in 1 second, SD = standard deviation, ICS = inhaled corticosteroids. * At baseline 94% of the children used ICS. ^†^ PC_20_: concentration of histamine inducing a 20% drop in FEV_1_. ^‡^ Atopy is defined as a positive Phadiatop (Phadia, Uppsala, Sweden), or a positive allergen skin test.

**Table 3:** Results of the multivariate models with the PAQLQ score as the dependent variable and independent predictors of asthma control (assessed by the ACQ) and other (clinical) parameters (dose of fluticasone or equivalent, age, sex, trial site, season, chronic rhinitis, wheezing episodes in the preceding year, PC_20_ histamine test, atopy, FeNO, exacerbations in the previous 2 months, and exposure to second-hand smoke). Only significant results are shown.

	PAQLQ _total score_			PAQLQ _symptoms domain_			PAQLQ _activity limitations domain_		
	β	CI	*P_value_*	β	CI	*P_value_*	β	CI	*P_value_*
ACQ asthma level of control^*^	0.43	0.37, 0.50	<0.01	0.63	0.54, 0.71	<0.01	0.60	0.50, 0.69	<0.01
**Fluticasone daily dosage or equivalent per 100 µg ^†^**	−0.04	−0.07, −0.008	0.01	−0.06	−0.10, −0.02	<0.01	−0.04	−0.08, −0.0004	0.05
**Season^‡^**	^‡^	^‡^	0.01			0.01	-	-	-
**Sex^§^**	-	-	-	-	-	-	−0.19	−0.36, −0.02	0.03

ACQ = Asthma Control Questionnaire, ICS = inhaled corticosteroids, PAQLQ = Pediatric Asthma Control Quality of Life Questionnaire, β = estimate of corresponding factor in the model, CI = 95% confidence interval of the estimate. The presented data are adjusted for trial site, age, chronic rhinitis, occurrence of wheezing episodes in the preceding year, PC_20_ histamine test, atopy, FeNO, exacerbation in the previous 2 months, and exposure to second-hand smoke. ^*^ Increase in the PAQLQ score for an improvement of ACQ asthma control from uncontrolled to partly controlled and partly controlled to controlled. ^†^ Decrease in the PAQLQ score per increase of 100 µg fluticasone daily dosage or equivalent. ^‡^ Decrease in the PAQLQ score for summer versus winter ((estimate) (95% CI) (−0.16) (−0.28, −0.04)) or summer versus spring ((estimate) (95% CI) (−0.17) (−0.28, −0.07)). ^§^ Decrease in the PAQLQ score for female versus male. Model with the PAQLQ emotional functioning domain as the dependent variable: of all factors, only the ACQ asthma level of control had a significant association (see text).

**Table 4:** Results of the multivariate models with the PAQLQ score as the dependent variable and independent predictors (asthma control according to GINA, dose of fluticasone or equivalent, age, sex, trial site, season, wheezing episodes in the preceding year, PC_20_ histamine test, atopy, FeNO, exacerbations in the previous 2 months, and exposure to second-hand smoke). Only significant results are shown.

	PAQLQ _total score_			PAQLQ _symptoms domain_			PAQLQ _activity limitations domain_		
	β	CI	*P_value_*	β	CI	*P_value_*	β	CI	*P_value_*
GINA asthma level of control^*^	0.38	0.30, 0.46	<0.01	0.57	0.47, 0.66	<0.01	0.56	0.44, 0.67	<0.01
**Wheezing episodes preceding year no/yes^†^**	−0.29	−0.10, −0.49	<0.01	−0.42	−0.16, −0.69	<0.01	−0.36	−0.05, −0.66	0.02
**Age in years^‡^**	-	-	-	−0.04	−0.08, <0.01	0.03	−0.05	−0.1, −0.01	0.02
**Exacerbation in previous 2 months no/yes^§^**	-	-	-	−0.19	−0.35, −0.02	0.03	-	-	-

GINA = Global Initiative for Asthma, PAQLQ = Pediatric Asthma Control Quality of Life Questionnaire, β = estimate of corresponding factor in the model, CI = 95% confidence interval of the estimate. Adjusted for trial site, sex, season, PC_20_ histamine test, atopy, FeNO, use of fluticasone or equivalent, and exposure to second-hand smoke. ^*^ Increase in the PAQLQ score for an increase of the level of control from uncontrolled to partly controlled to controlled based on the GINA criteria. ^†^ Decrease in the PAQLQ score in the case of wheezing episodes having occurred in the preceding year. ^‡^ Decrease in the PAQLQ score when age increase. ^§^ Decrease in the PAQLQ score in the case of the occurrence of exacerbations in the preceding 2 months. Model with the PAQLQ emotional functioning domain as the dependent variable: of all factors, only the GINA asthma level of control had a significant association (see text).

**Table 5:** Results of longitudinal associations between the PAQLQ scores and FEV_1_ percentage of the predicted value, β_2_-agonist use, dose of fluticasone or equivalent, age, sex, trial site, season, wheezing episodes in the preceding year, PC_20_ histamine test, atopy, FeNO, exacerbations in the previous 2 months, and exposure to second-hand smoke. Only data of significant predictors are shown and asthma control was excluded from the analysis.

	PAQLQ _total score_			PAQLQ _symptoms domain_			PAQLQ _activity limitations domain_		
	β	CI	*P_value_*	β	CI	*P_value_*	β	CI	*P_value_*
FEV_1_ per 10% predicted value *	0.05	0.01, 0.08	<0.01	0.08	0.03, 0.12	<0.01	0.06	0.00, 0.11	0.04
**β_2_-agonist use ^†^**	−0.25	−0.32, −0.18	<0.01	−0.40	−0.49, −0.31	<0.01	−0.39	−0.49, 0.28	<0.01
**Exacerbation previous 2 months no/yes ^‡^**	−0.18	−0.30, −0.07	<0.01	−0.36	−0.51, −0.20	<0.01	-	-	-
**Wheezing episodes preceding year no/yes ^§^**	−0.23	−0.06, −0.41	0.01	−0.30	−0.06, −0.54	0.02	−0.27	−0.01, −0.53	0.04

FEV_1_: forced expiratory volume in 1 second, β_2_-agonist = use of rescue medication, PAQLQ = Pediatric Asthma Control Quality of Life Questionnaire, β = estimate of corresponding factor in the model, CI = 95% confidence interval of the estimate. Adjusted for: seasons, trial site (Maastricht or Sittard), sex, age, PC_20_ histamine test, atopy, exposure to second-hand smoke, FeNO, and daily dose of fluticasone or equivalent. * Increase in the PAQLQ score/10% increase of the FEV_1_ percentage of the predicted value. ^†^ Decrease in the PAQLQ score/increase in the ACQ score concerning a β_2_-agonist use. **^‡^** Decrease in the PAQLQ score in the case of the occurrence of exacerbations in the preceding 2 months. ^§^ Increase in the PAQLQ score in the case of increasing wheezing episodes during the year preceding the study. Model with the PAQLQ emotional functioning domain as the dependent variable: no factor was associated with the PAQLQ emotion functioning domain (see text).
